# On the presence and role of human gene-body DNA methylation

**DOI:** 10.18632/oncotarget.497

**Published:** 2012-05-09

**Authors:** Daudi Jjingo, Andrew B. Conley, Soojin V. Yi, Victoria V. Lunyak, I. King Jordan

**Affiliations:** ^1^ School of Biology, Georgia Institute of Technology, Atlanta GA, 30332 USA; ^2^ Buck Institute for Research on Aging, Novato CA, 94945 USA; ^3^ PanAmerican Bioinformatics Institute, Santa Marta, Magdalena, Colombia

**Keywords:** genome-wide methylation, epigenetic mark, intragenic transcription, methylating enzyme complexes

## Abstract

DNA methylation of promoter sequences is a repressive epigenetic mark that down-regulates gene expression. However, DNA methylation is more prevalent within gene-bodies than seen for promoters, and gene-body methylation has been observed to be positively correlated with gene expression levels. This paradox remains unexplained, and accordingly the role of DNA methylation in gene-bodies is poorly understood. We addressed the presence and role of human gene-body DNA methylation using a meta-analysis of human genome-wide methylation, expression and chromatin data sets. Methylation is associated with transcribed regions as genic sequences have higher levels of methylation than intergenic or promoter sequences. We also find that the relationship between gene-body DNA methylation and expression levels is non-monotonic and bell-shaped. Mid-level expressed genes have the highest levels of gene-body methylation, whereas the most lowly and highly expressed sets of genes both have low levels of methylation. While gene-body methylation can be seen to efficiently repress the initiation of intragenic transcription, the vast majority of methylated sites within genes are not associated with intragenic promoters. In fact, highly expressed genes initiate the most intragenic transcription, which is inconsistent with the previously held notion that gene-body methylation serves to repress spurious intragenic transcription to allow for efficient transcriptional elongation. These observations lead us to propose a model to explain the presence of human gene-body methylation. This model holds that the repression of intragenic transcription by gene-body methylation is largely epiphenomenal, and suggests that gene-body methylation levels are predominantly shaped via the accessibility of the DNA to methylating enzyme complexes.

## INTRODUCTION

DNA methylation is a crucial epigenetic mark with roles in embryogenesis and differentiation [1], X-inactivation [2], imprinting [3] and repression of viral and repeat sequences [4]. Changes in patterns of DNA methylation have been implicated in the pathogenesis of several human diseases [5, 6] including cancer [7]. One long established role of DNA methylation in promoter regions is the repression of transcription [1, 8, 9]. As a result, methylation is largely depleted from the promoter regions of genes. In contrast, DNA methylation in gene bodies is surprisingly abundant and has been reported to show a positive correlation with gene expression [10-15] even though it can interfere with transcription elongation [16]. The apparent contradiction between the activities of DNA methylation in promoters versus gene bodies has been referred to as the DNA methylation paradox [17]. Here, we address this paradox in an effort to better understand the presence and role of DNA methylation in human gene bodies.

Repression of spurious transcription within genes is one possible explanation for the prevalence of gene-body methylation. Indeed, relatively low average levels of DNA methylation genome-wide have been taken to suggest that the primary role of methylation is the repression of spurious transcription rather than the regulation of promoters *per se* [18]. More recently, Cap Analysis of Gene Expression (CAGE) data have confirmed that transcription is very frequently initiated from within genes, albeit at lower levels than seen for canonical 5’ gene promoters [19, 20]. Thus, it is reasonable to assume that there may be some need to repress this intragenic transcription. Repression of intragenic promoters by DNA methylation could allow for more efficient transcriptional elongation, thus accounting for the reported positive correlations between gene expression and gene-body methylation levels.

This model predicts a negative correlation between levels of gene-body methylation and the initiation of intragenic transcripts. Such a negative correlation was recently shown for the case of the human SHANK3 locus where intragenic methylation regulates intragenic promoter activity [20]. This same study showed that within intragenic CpG islands genome-wide, there is an overall negative correlation between transcription initiation and methylation levels. Nevertheless, the extent to which this relationship holds across gene-bodies is unclear since there are numerous CpG sites and promoters outside of CpG islands [21].

The notion that gene-body methylation serves to repress intragenic transcription, thereby allowing for more efficient transcriptional elongation also rests on the reported clear and monotonic positive correlations observed between gene expression levels and gene-body methylation[11-15]. However, the relationship between gene-body methylation and expression levels appears to be more complicated than previously imagined. In some plants and invertebrates, the relationship is not monotonic but rather bell shaped with genes expressed at the mid-range levels having the highest methylation levels [22, 23]. More recently, when a variety human tissue types were analyzed, some showed a monotonic positive correlation between expression and gene-body methylation whereas others showed no apparent relationship [10]. Thus, it remains uncertain whether repression of spurious intragenic transcription best explains the high levels of observed gene-body DNA methylation.

Here, we revisit this issue taking advantage of the recent accumulation of genome-scale datasets provided by the ENCODE [24, 25] and RIKEN groups. In particular, the availability of genome-wide human methylation [26], expression [19, 27-30] and chromatin datasets [31, 32] provide deep resolution for an interrogation of the DNA methylation paradox. Meta-analysis of these genome-scale data sets revealed that 1) the relationship between gene-body DNA methylation and gene expression is non-monotonic rather than linear, and 2) while gene-body DNA methylation does serve to repress spurious transcription, that role does not explain the majority of methylation in gene-bodies. These results suggest a model whereby gene-body DNA methylation is chiefly determined by DNA accessibility to methylating enzymes during transcription, and the repression of intragenic transcription is simply an epiphenomenal byproduct of this process. The model accounts for the majority of gene-body methylation, which cannot be explained by the need to repress spurious transcription alone. It also explains the observed non-monotonic relationship between gene-body DNA methylation and gene expression.

## RESULTS

### Meta-analysis of genome-wide methylation, expression and chromatin data sets

The ENCODE project has generated a rich collection of elements that associate with DNA sequences and have functional consequences for the way the genome is regulated. For this study, we made use of four datasets from the ENCODE project: 1) DNA methylation data generated by RRBS[26], 2) gene expression data generated from human exon microarrays[27, 28], 3) RNA polymerase II (Pol2) binding locations generated by ChIP-Seq [31, 33-36] and 4) the genomic locations of DNaseI hypersensitive sites (DHSS) generated by the digital DNaseI technique [32]. Additionally, we used a fifth dataset from the RIKEN Omics Science center made up of CAGE tags that characterize the 5’ ends of full-length transcripts [29]. All five of these datasets were available for three cell-lines (GM12878, K562 and HepG2), which together entail the primary focus of the study, and different subsets of the same five datasets were available in three additional cell-lines (HeLa-S3, H1hESC and HUVEC) (Table 1). These datasets were analyzed in various combinations across cell-lines in order to interrogate specific aspects of the relationship between DNA methylation, chromatin and gene expression.

**Table 1:** Genome-wide expression and chromatin datasets analyzed in this study

Measure^a^	Technique^b^	Cell Types^c^	GEO Accessions^d^	PMID^e^
**DNA methylation**	Reduced representation bisulphite sequencing	GM12878 K562 HepG2 HeLa-S3 H1hESC	GSE27584	18600261
**Gene expression**	Exon microarray	GM12878 K562 HepG2 HeLa-S3 H1Hesc HUVEC	GSE19090	19966280
**Intragenic transcription initiation**	Cap analysis of gene expression (CAGE)	GM12878 K562 HepG2	N/A	16489339 8938445 19074369
**RNA Pol2 binding density**	Chromatin immunoprecipitation followed by high-throughput sequencing (ChIP-Seq)	GM12878 K562 HepG2 HeLa-S3 H1Hesc HUVEC	GSE32465	17556576 17540862 19160518 18798982
**DNaseI hypersensitive site density**	Digitial DNaseI	GM12878 K562 HepG2 HeLa-S3 H1Hesc HUVEC	GSE8962 GSE7411	15550541 16791208

a Specific aspect of gene expression or chromatin being measured

b Experimental technique or assay used

c ENCODE cell types for which the data are available

d Gene Expression Omnibus (GEO) accession numbers for the data

e PubMed IDs (PMID) for the references reporting the data

### A non-monotonic relationship between gene-body methylation and human gene expression

The DNA methylation paradox is borne of the fact that in human promoter regions CpG methylation is negatively correlated to gene expression levels, while in gene bodies CpG methylation is apparently positively correlated to gene expression [18]. Furthermore, recent genome-scale analyses of human methylation and gene expression suggest that this relationship is monotonic, *i.e.* gene-body methylation levels rise consistently across increasing intervals of gene expression [11, 13-15].

**Figure 1:**
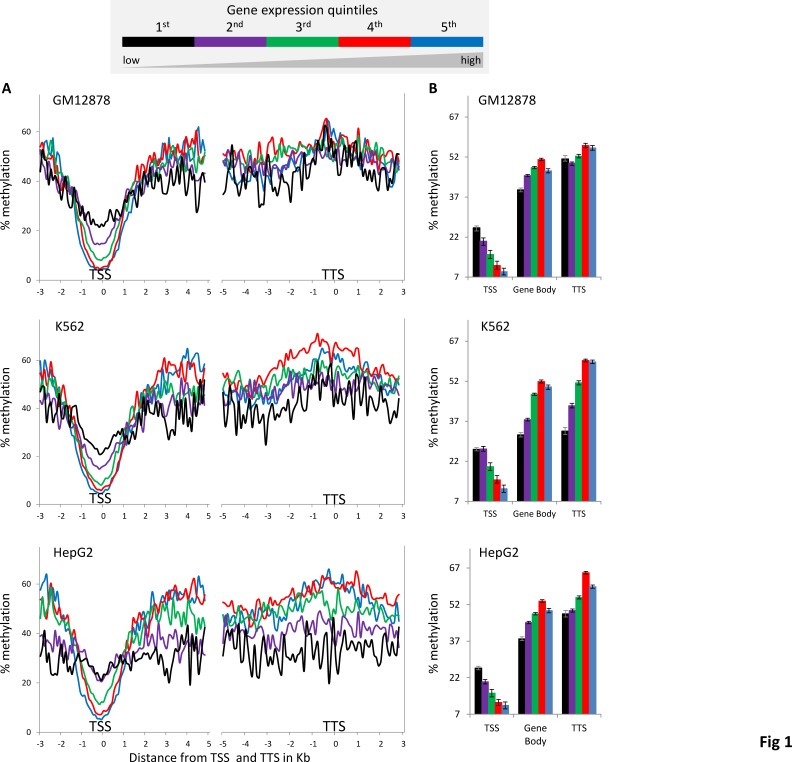
DNA methylation levels around the TSS, gene-body and TTS across five gene expression level bins (A) Average percentage methylation levels of 100bp windows spanning the TSS, gene-body and TTS, showing 3kb and 5kb upstream and downstream of TSS respectively and 5kb and 3kb upstream and downstream of TTS respectively. (B) Overall average (± standard error) percentage methylation levels for TTS, gene-body and TTS.

We further evaluated this paradoxical relationship using DNA methylation and gene expression data from ENCODE cell-lines (Table 1). To do this, percent DNA methylation values in-and-around gene-bodies were compared across five gene expression level quintiles. Consistent with previous reports in human cell-lines [11, 13], DNA methylation levels around transcription start sites (TSS) at the 5’ ends of genes show a clearly negative and monotonic correlation with gene expression levels (Figure 1 and Supplementary Material, Figure S1). The TSS regions of highly expressed genes are relatively depleted for DNA methylation whereas genes expressed at lower levels are increasingly methylated.

However, the relationship between gene-body methylation and expression levels is different from what has been described before; gene-body methylation levels show a bell-shaped, rather than monotonic, relationship with gene expression levels (Figure 1 and Supplementary Material, Figure S1). Generally, mid-level expressed genes in the 3^rd^ and 4^th^ quintiles have the highest DNA methylation percentages while those in the 2^nd^ and 5^th^ quintiles show medium DNA methylation percentages and those in the 1^st^ quintile show the lowest DNA methylation percentages. A similar bell-shaped relationship between gene-body methylation and expression levels has been observed previously in plants (*Arabidopsis thaliana* and *Oryza sativa*) and invertebrates (*Ciona intestinalis* and *Nematostella vectensis*) [22, 23]. Human gene-body methylation levels measured here are about the same as those of the TTS regions but higher than those seen for both the regions surrounding TSS and the associated intergenic regions (Figure 1 and Supplementary Material, Figure S1).

**Figure 2:**
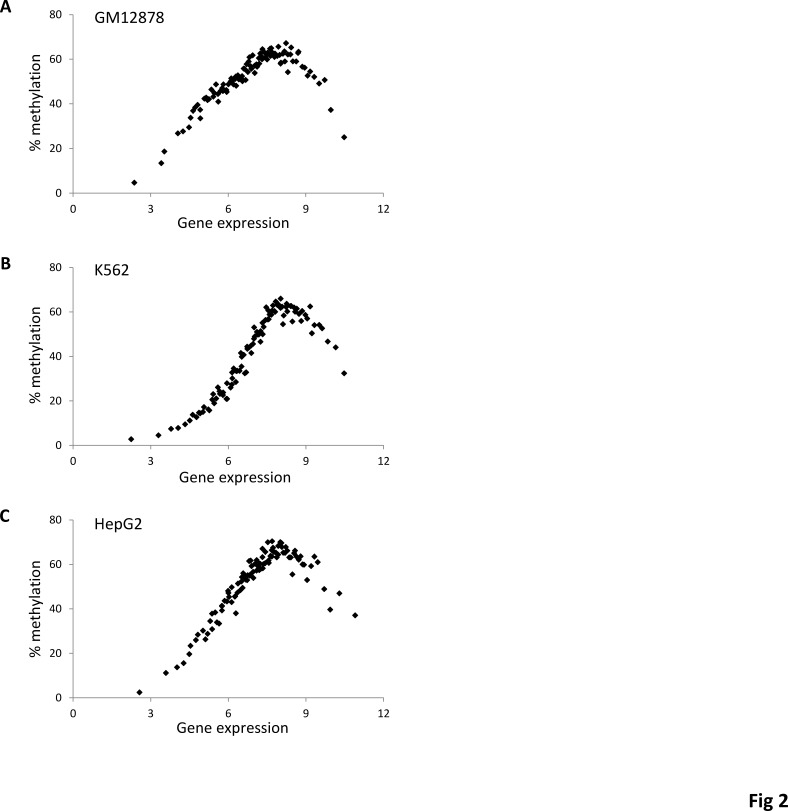
A non-monotonic relationship between gene-body DNA methylation and gene expression Overall percentage methylation of gene-bodies (regions starting at 1kb downstream of the TSS and ending at 1kb upstream of the TTS of genes) is regressed against gene expression for (A) GM12878 (B) K562 and (C) HepG2. Genes are grouped into 100 gene expression bins.

In light of the unexpected but distinct non-monotonic relationship for human gene-body methylation and gene expression observed here, we sought to evaluate this pattern at a higher level of resolution. To do this, human genes were divided into 100 expression level bins, and then methylation and gene expression levels were regressed across these intervals. This analysis further revealed a clearly non-monotonic and bell-shaped relationship between gene-body methylation and gene expression in all five human cell lines for which methylation data was available (Figure 2 and Supplementary Material, Figure S2). The mid-level expressed genes showed the highest DNA methylation levels while both the lowest and highest expressed genes had markedly lower DNA methylation levels.

DNA methylation levels have also been found to be related to gene length [37]. We thus sought to check if the bell-shaped relationship we found between gene-body methylation and gene expression is not infact a reflection of the relationship between DNA methylation and gene length. To do this, we checked if the bell-shaped relationship would still be present for sets of genes with widely differing lengths. We found a similar bell-shaped non-montonic relationship between gene-body methylation and gene expression for both the 20% shortest and 20% longest genes suggesting that the relationship is independent of gene length (Supplementary Material, Figure S3).

### Gene-body methylation represses the initiation of intragenic transcription

DNA methylation was originally thought to serve primarily to repress spurious transcription [18], and gene-body methylation has been shown to repress the activity of intragenic promoters [20]. Thus, it may be the case that gene-body methylation serves to repress spurious transcription from intragenic promoters, thereby allowing for more efficient transcriptional elongation. This kind of repressive role for DNA methylation could explain the relative abundance of DNA methylation within gene-bodies and its reported positive correlation with gene expression.

**Figure 3:**
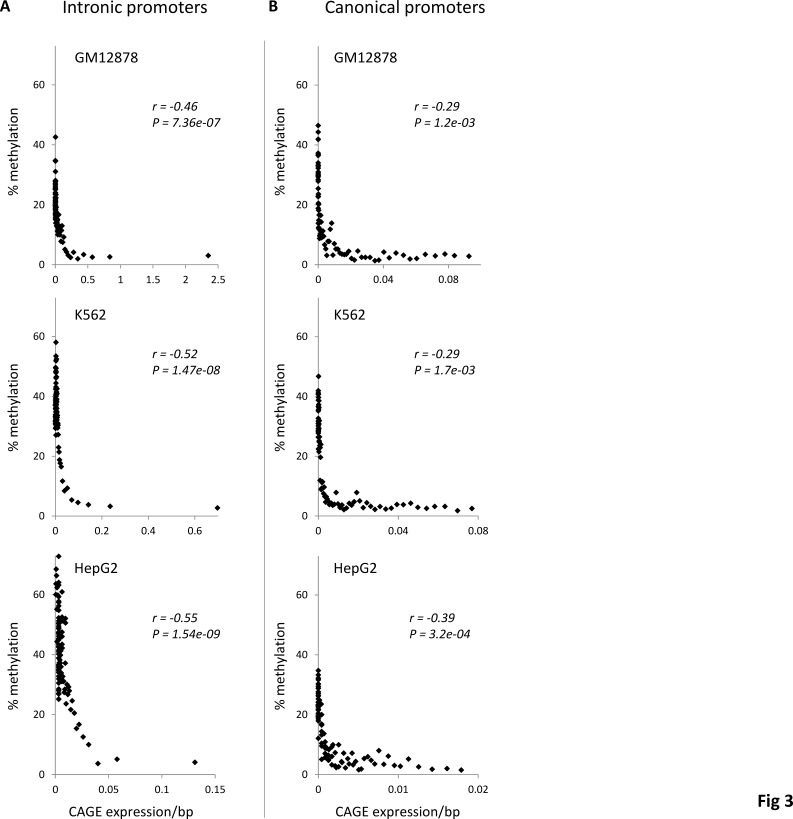
Relationship between DNA methylation and promoter activity levels Percent DNA methylation levels are regressed against CAGE expression levels (*i.e.* promoter activity) for (A) intronic and (B) canonical 5’ gene promoters. Genes are grouped into 100 gene expression bins. Pearson correlation coefficient values (*r*) along with their significance values (*P*) are shown for each regression.

To evaluate this possibility here, we used CAGE data to analyze the relationship between gene-body methylation and the repression of intragenic transcription. Intronic CAGE clusters mark intragenic promoters and the levels of transcriptional initiation from these intragenic promoters are characterized by the number of CAGE tags per intronic cluster [19, 20]. We mapped intragenic promoters across three ENCODE cell-lines using CAGE, and then DNA methylation levels at these intragenic promoters were regressed against the promoter activity levels measured by CAGE tag density. For all three cell-lines, this analysis revealed significantly negative correlations between the DNA methylation levels of intronic promoters and their corresponding transcriptional initiation levels (Figure 3A). These data are consistent with the repression of intragenic promoters by DNA methylation. Indeed, a similar analysis of canonical TSS from the 5’ ends of the genes, where the repressive role of DNA methylation is well known, yields qualitatively identical results (Figure 3B).

### Gene-body methylation, transcription and open chromatin

**Figure 4:**
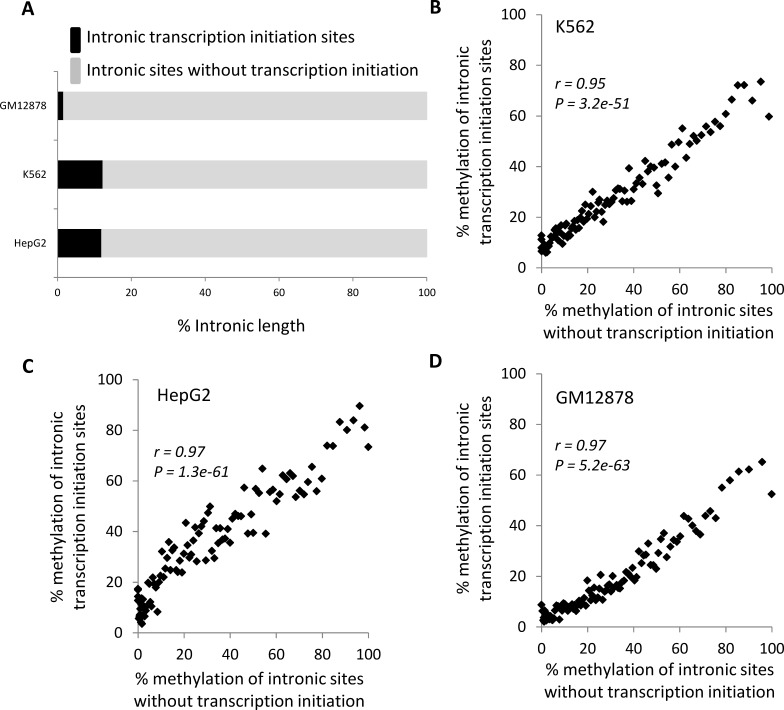
Comparison of length and DNA methylation attributes of intronic promoters and intronic sites without transcription initiation (A) Percentage of intronic length occupied by transcription initiation sites (black) and versus sites without transcription initiation (grey). Percent DNA methylation levels for transcription initiation sites are regressed against methylation levels for non-transcription initiation sites for (B) GM12878, (C) K562 and (D) HepG2 cell-lines. Genes are grouped into 100 methylation level bins. Pearson correlation coefficient values (*r*) along with their significance values (*P*) are shown for each regression.

Results from the previous section indicate that gene-body methylation can repress intragenic transcription. Accordingly, if the primary role of gene-body methylation is to repress spurious intragenic transcription, then there should be more DNA methylation at intronic promoters than at intronic sites that do not initiate transcription. However, we find the vast majority of gene-body DNA methylation maps to sites that do not initiate transcription (Figure 4A). Presumably, this majority fraction of intronic DNA methylation does not serve to repress transcription. Furthermore, levels of gene-body methylation are highly positively correlated for these two classes of intronic sites: transcriptional initation sites and non-transcriptional initiation sites (Figure 4B-D). In other words, there is no particular enrichment of DNA methylation at intragenic promoters compared to their surrounding genic environment. Rather, DNA methylation levels are consistent across introns of individual gene-bodies and appear to be largely determined by something other than the need to repress intragenic transcription.

**Figure 5:**
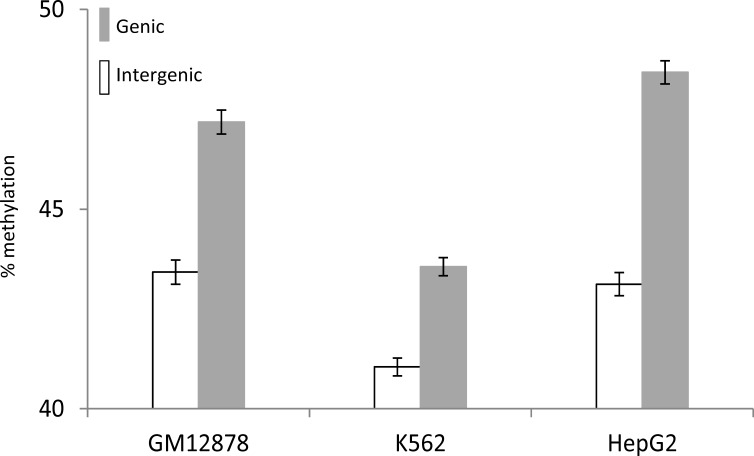
Comparison between genic and intergenic average (± standard error) DNA methylation levels in GM12878, K562 and HepG2 cell-lines

These results instead suggest that gene-body DNA methylation is deposited onto introns by a mechanistically independent process, and that only a small fraction of the DNA methylated sites are involved in the silencing of spurious intragenic transcription. The relationship we observe between gene-body DNA methylation and gene expression (Figure 2) suggests that the transcriptional elongation process, together with its associated open chromatin, might account for much of gene-body methylation. If gene-body methylation is linked to transcriptional elongation, then transcribed regions would have higher levels of DNA methylation relative to un-transcribed regions. In fact, we observe that human genic regions do have substantially higher levels of DNA methylation than seen for intergenic regions (Figure 5 and Supplementary Material, Figure S4). In addition, a similar elevation of DNA methylation levels for transcribed genic regions has been reported in a number of other species [22, 38].

**Figure 6:**
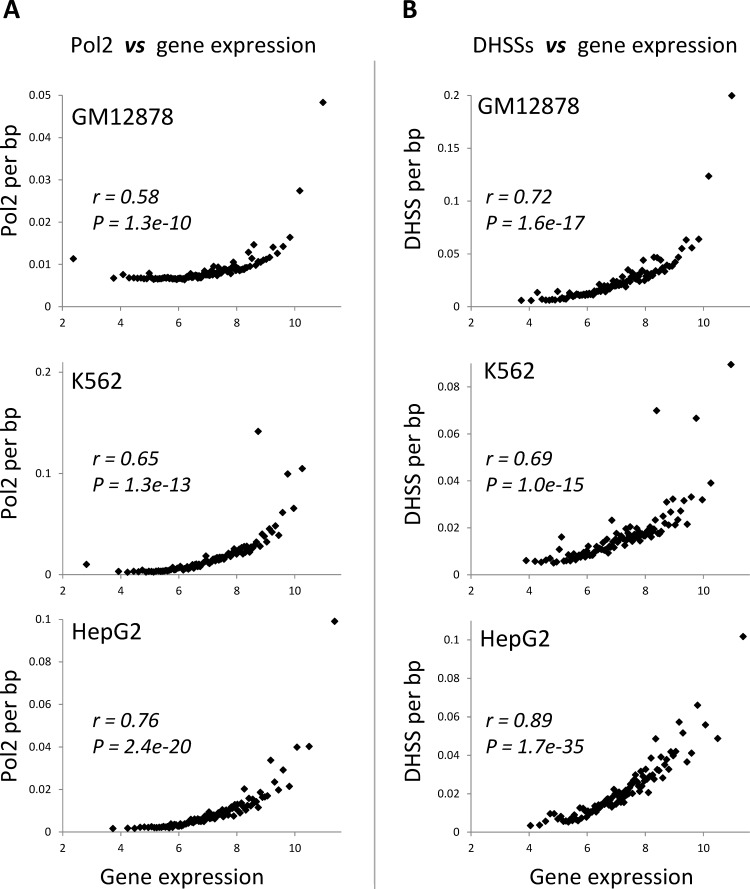
Relationship between chromatin environment and gene expression levels (A) Pol2 occupancy and (B) density of DHSS sites are regressed against gene expression. Genes are grouped into 100 gene expression bins. Pearson correlation coefficient values (*r*) along with their significance values (*P*) are shown for each regression.

DNA methylation is clearly associated with the presence of transcribed gene regions, and levels of transcription for these gene regions are expected to be associated with a distinct chromatin environment including high occupancy levels of Pol2 and the presence of demonstrably open chromatin. To test this, we regressed gene expression levels against Pol2 occupancy levels and the extent of open chromatin measured by the presence of DNaseI hypersensitive sites (DHSS). Both Pol2 occupancy levels and the extent of open chromatin are in fact highly positively correlated with gene expression across all six ENCODE cell-lines evaluated here (Figure 6 and Supplementary Material, Figure S5).

When considered together with the data showing that gene-body methylation accumulates independent of the need to repress spurious intragenic transcription (Figure 4), these results suggest that the presence of open chromatin *per se* is an important prerequisite for the deposition of gene-body methylation. However, the relationship between gene-body methylation and open chromatin is non-monotonic, suggesting that the extent of open chromatin alone does not determine gene-body methylation levels. In the discussion section, we propose a specific model to explain the presence of gene-body DNA methylation that accounts for this complexity.

## DISCUSSION

DNA methylation is a well known repressive chromatin mark when associated with promoter regions. However, DNA methylation is far more prevalent in gene-bodies than in promoters and the role of gene-body methylation is still not clearly understood. In this study, we performed a meta-analysis of genome-wide methylation, expression and chromatin data sets in an attempt to better understand the presence and role of gene-body DNA methylation.

We show that levels of DNA methylation are more clearly related to the presence of transcribed regions than to the impetus to repress spurious intragenic transcription. However, the quantitative relationship between gene-body methylation and expression levels in non-monotonic and bell-shaped. On the other hand, the relationships between gene expression levels and Pol2 occupancy along with open chromatin are positive and monotonic. Considered together, these results link gene-body methylation to transcription and open chromatin, albeit in a complex and non-linear way. Here, we propose a specific model to explain the presence of gene-body DNA methylation in light of these results.

Our model rests on the notion that the deposition of DNA methylation is mechanistically facilitated, to some extent, by open and actively transcribed chromatin. In support of this contention, a biochemical study demonstrated that DNA methyltransferase 1 (DNMT1) interacts with Pol2 by binding the C-terminal repeat domain of Pol2 [39]. It has also been shown that the catalytic domain of DNMT1 needs to directly bind to DNA and to transit along the DNA molecule in order to function [40, 41]. Nevertheless, the bell-shaped relationship between gene-body methylation and expression levels indicates that open and actively transcribed chromatin does not completely determine gene-body methylation. On the contrary, there appears to be some trade-off between the openness of the chromatin and the levels of DNA methylation, and we also try to account for this in our model.

**Figure 7:**
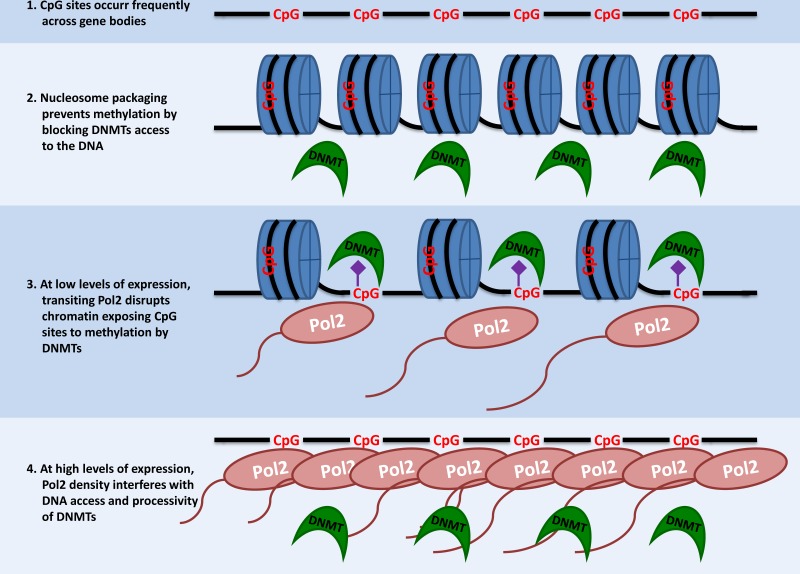
Model showing how interactions between chromatin openness and Pol2 density specify gene-body DNA methylation DNA (black string), CpG sites (potential methylation sites - red), methyl groups (purple), nucleosomes (blue), polymerases (brown) and DNMT1(green).

The model explaining levels of gene-body methylation is illustrated in Figure 7 and can be summarized as follows. The extent of nucleosome packaging seen for unexpressed and compact chromatin would not allow for access to the DNA by DNMT1, effectively blocking DNA methylation. At low levels of transcription, transiting Pol2 complexes disrupt nucleosome packaging and open up the chromatin thereby exposing CpG sites for methylation. Therefore, levels of gene-body methylation will increase with increasing levels of expression at the low end of the expression spectrum. However, as genes become increasingly highly expressed, the density of transiting Pol2 becomes so high as to begin to interfere with the processivity of DNMT1 along DNA. This leads to a progressive reduction of gene-body methylation levels with increasing expression levels at the high end of the expression spectrum. Therefore, the most lowly and the most highly expressed genes will have the lowest levels of methylation, whereas genes expressed at intermediate levels will have the highest gene-body methylation, as seen here for humans and elsewhere for other species [22, 23].

**Figure 8:**
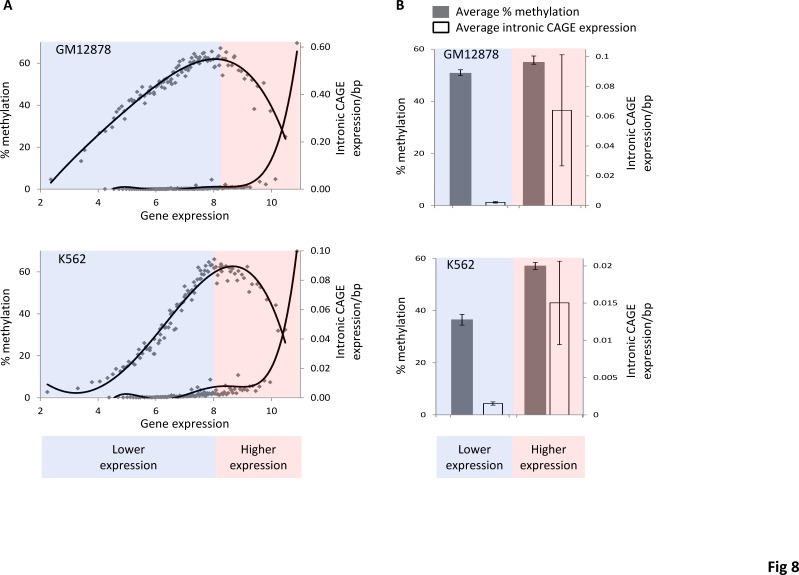
Decreasing levels of gene body methylation, starting from mid-levels of gene expression are correlated with increasing levels of intronic expression Highly expressed genes are represented by pink background while lowly expressed genes are represented by light blue background. (A) Gene expression levels are regressed against percent gene-body methylation (top curve) and levels of intronic expression (bottom curve). (B) Comparison of average intronic transcription (open bars) and average percentage methylation (grey bars) between lowly and highly expressed genes.

While we find this model to be mechanistically compelling for the reasons described above, it does not directly address the demonstrated role of gene-body DNA methylation in repressing spurious intragenic transcription. To investigate this further, we re-evaluated the intronic CAGE data in light of the non-monotonic relationship between gene expression and gene-body methylation levels. Regressing intronic CAGE levels against gene expression data and comparing this relationship to that seen for methylation and expression reveals a coincident inflection point between the two curves where methylation levels fall off to such an extent as to begin to allow for the initiation of transcription from intragenic promoters (Figure 8). This observation unites the DNA accessibility model for gene-body methylation that we propose with the role of methylation in repressing intragenic transcription. However, the juxtaposition of these two phenomena can also be taken to suggest the intriguing possibility that the observed repression of intragenic transcription by methylation is simply a by-product of relative accessibility levels to the DNA by methylating enzymes.

The relationship between gene expression levels, Pol2 density and initiation of transcription from intragenic promoters also serves to distinguish our observations and model from what has previously been proposed for *A. thaliana* [23]. The *A. thaliana* model also attempted to explain an observed bell-shaped distribution for gene-body methylation with respect to expression, and the model held that gene-body methylation was facilitated by the transcription of siRNAs from intragenic promoters. Transcription of these intragenic siRNAs was thought to be facilitated by the progressive opening of the chromatin from low-to-mid levels of expression, and then these siRNAs would interact with their cognate DNA sequences to attract the methylation machinery *in situ*. However, at high levels of transcription, Pol2 density was thought to be too great to allow for the initiation of intragenic transcription thus accounting for the low levels of methylation for highly expressed genes. On the contrary, here we observe that the initiation of transcription from intragenic promoters increases steadily with increasing expression and Pol2 occupancy levels peaking among highly expressed genes that also show low levels of gene-body methylation (Figure 8).

It should also be noted that our observations on the relationship between expression level and gene-body methylation, at the high end of expression, are consistent with previous results showing that gene-body methylation interferes with transcriptional elongation [16]. Thus, the patterns observed here may also point to incompatibility and selection against high levels of gene-body methylation for highly expressed genes.

## MATERIALS AND METHODS

### Human gene loci

Gene annotations for the March 2006 build of the human genome reference sequence (NCBI build 36.1; UCSC hg18) were taken from the ‘RefSeq Genes’ track of the UCSC Genome Browser [42, 43]. Individual genes were defined as distinct genomic loci encompassing all overlapping RefSeq transcripts from the start of the 5’ most exon to the end of the 3’ most exon. A total of 32,128 RefSeq transcripts were merged into 19,539 genes that represent distinct gene loci.

### DNA methylation

Genome-wide DNA methylation data for the GM12878, K562, HepG2, HeLa-S3 and H1Hesc cell-lines were taken from the ‘ENCODE DNA methylation track’ of the UCSC Genome Browser (assembly hg19). Methylation data were generated using the Reduced Representation Bisulfite Sequencing (RRBS) technique [26] and cover approximately 1.26-1.47 million CpG sites in each of the five cell-lines. The RRBS methylation data are represented as percent methylation for each covered CpG site, and herein DNA methylation levels for any locus or genomic region were computed as the average percentage methylation of all cytosine residues covered therein.

### Gene expression

Exon microarray data for six ENCODE cell-lines (GM12878, K562, HepG2, HeLa-S3, H1Hesc and HUVEC) were taken from the ‘ENCODE Exon Array’ track of the UCSC Genome Browser (assembly hg19) [19, 27-30]. The data were generated using the Affymetrix Human Exon 1.0 ST GeneChip and analyzed using Affymetrix ExACT 1.2.1 software with samples quantile normalized using the PM-GCBG background correction and PLIER (probe logarithmic intensity error) summary. Here, the log_2_ normalized average signal intensity of all exons mapping to an individual gene locus was taken to represent the overall expression of the gene. This resulted into a final set of 18,632 genes for which expression data was available in all cell-lines.

Cap Analysis of Gene Expression (CAGE) data [29, 30, 44] were taken from the ‘RIKEN CAGE Loci’ track of the UCSC Genome Browser (assembly hg18). Nucleus CAGE clusters for GM12878 (1.18 million), K562 (8.86 million) and HepG2 (5.89 million) cell-lines were analyzed here. Discretely located CAGE clusters were taken as individual proximal promoters (or TSS), and promoter expression levels were computed as the number of CAGE tags in a cluster divided by the length of the cluster. Intronic CAGE expression levels were calculated in the same way over entire gene loci.

### RNA Polymerase II (Pol2)

RNA Polymerase II (Pol2) binding site ChIP-seq data [31, 33-36] were taken from the ‘HAIB TFBS’ track of the UCSC Genome Browser (assembly hg18). The ChIP-seq reads were re-mapped to the human genome reference sequence (assembly hg18) in order to rescue individual tags that map to multiple genomic locations as previously described [45], resulting in approximately 18.78, 6.78, 13.86, 6.78, 20.84, 22.61 and 12.34 million reads in the GM12878, K562, HepG2, HeLa-S3, H1Hesc and HUVEC cell-lines respectively. For each locus, Pol2 binding density was computed as the number of tags mapping on the locus, divided by the length of the locus.

### DNaseI Hypersensitive Sites (DHSS)

DNaseI Hypersensitive Site (DHSS) data, generated using the digital analysis of chromatin structure (DACS) technique [32], were taken from the ‘UW DNaseI HS’ track of the UCSC Genome Browser (assembly hg18). The DACS sequence reads were re-mapped to the human genome reference sequence (assembly hg18) in order to rescue individual tags that map to multiple genomic locations as previously described [45], resulting in approximately 30.40, 35.15, 27.32, 44.10, 28.59 and 38.40 million reads in the GM12878, K562, HepG2, HeLa-S3, H1Hesc and HUVEC cell-lines respectively. For each locus, DHSS density was computed as the number of tags mapping on the locus divided by the length of the locus.

## FUNDING

This work was supported in part by the Fulbright foundation through a PhD scholarship to DJ; The School of Biology, Georgia Institute of Technology [to IKJ, DJ and ABC]; An Alfred P. Sloan Research Fellowship in Computational and Evolutionary Molecular Biology [grant number BR-[4839] to IKJ]; the Buck Institute Trust Fund [to VVL]; National Science Foundation grants MCB-0950896 and BCS-0751481 [to SVY].

## Supplementary Figures


