# Supplementary material for: On the presence and role of human gene-body DNA methylation
Source: Oncotarget. 2012 May 9;3(4):462–74. doi: 10.18632/oncotarget.497 (PMC3380580; doi:10.18632/oncotarget.497)
Supplement: Supplementary file 1 [file oncotarget-03-462-s001.pdf]

Supplementary Material

On the presence and role of human gene-body DNA methylation

Daudi Jjingo, Andrew B. Conley, Soojin V. Yi, Victoria V. Lunyak and I. King Jordan

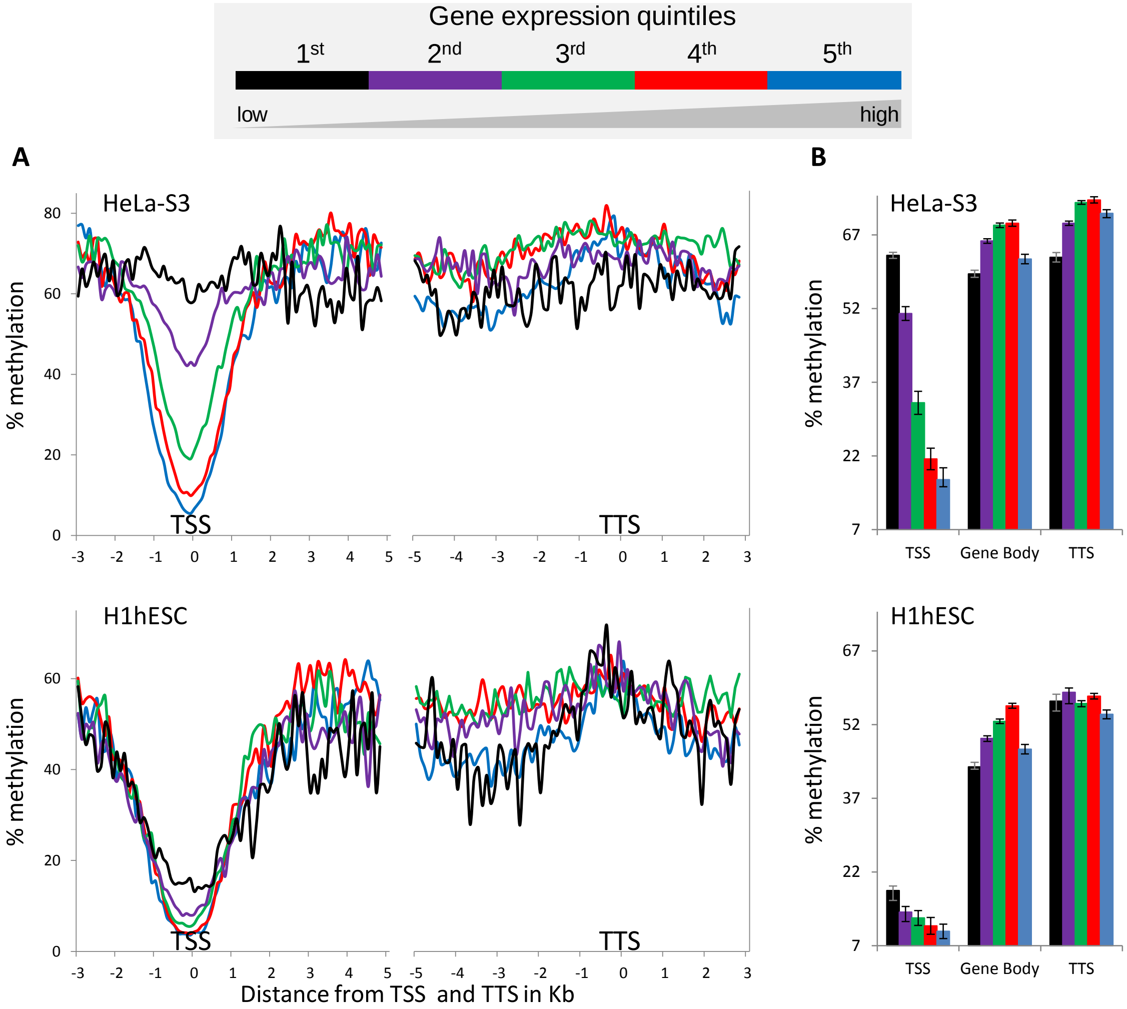

Supplementary Figure S1 – Gene expression-based percentage DNA methylation around the TSS, gene-body and TTS. (A) Average percentage methylation levels of 100bp windows spanning the TSS, gene-body and TTS, showing 3kb and 5kb upstream and downstream of TSS respectively and 5kb and 3kb upstream and downstream of TTS respectively. (B) Overall percentage methylation levels of groups of genes binned by expression. Error bars are standard errors.

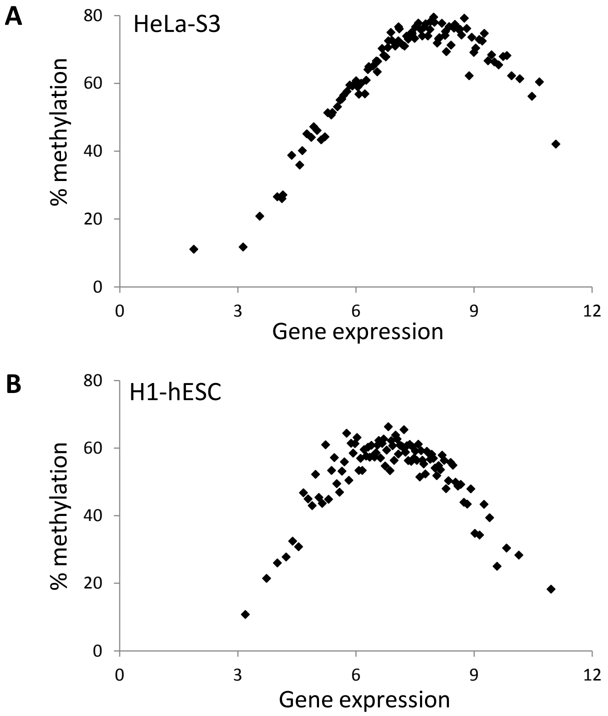

Supplementary Figure S2 – A non-monotonic relationship between gene-body DNA methylation and gene expression. Shows overall percentage methylation of gene-bodies (regions starting at 1kb downstream of the TSS and ending at 1kb upstream of the TTS). Each data point represents the average methylation and corresponding average expression of each bin of genes. (A) HeLa-S3. (B) H1-hESC.

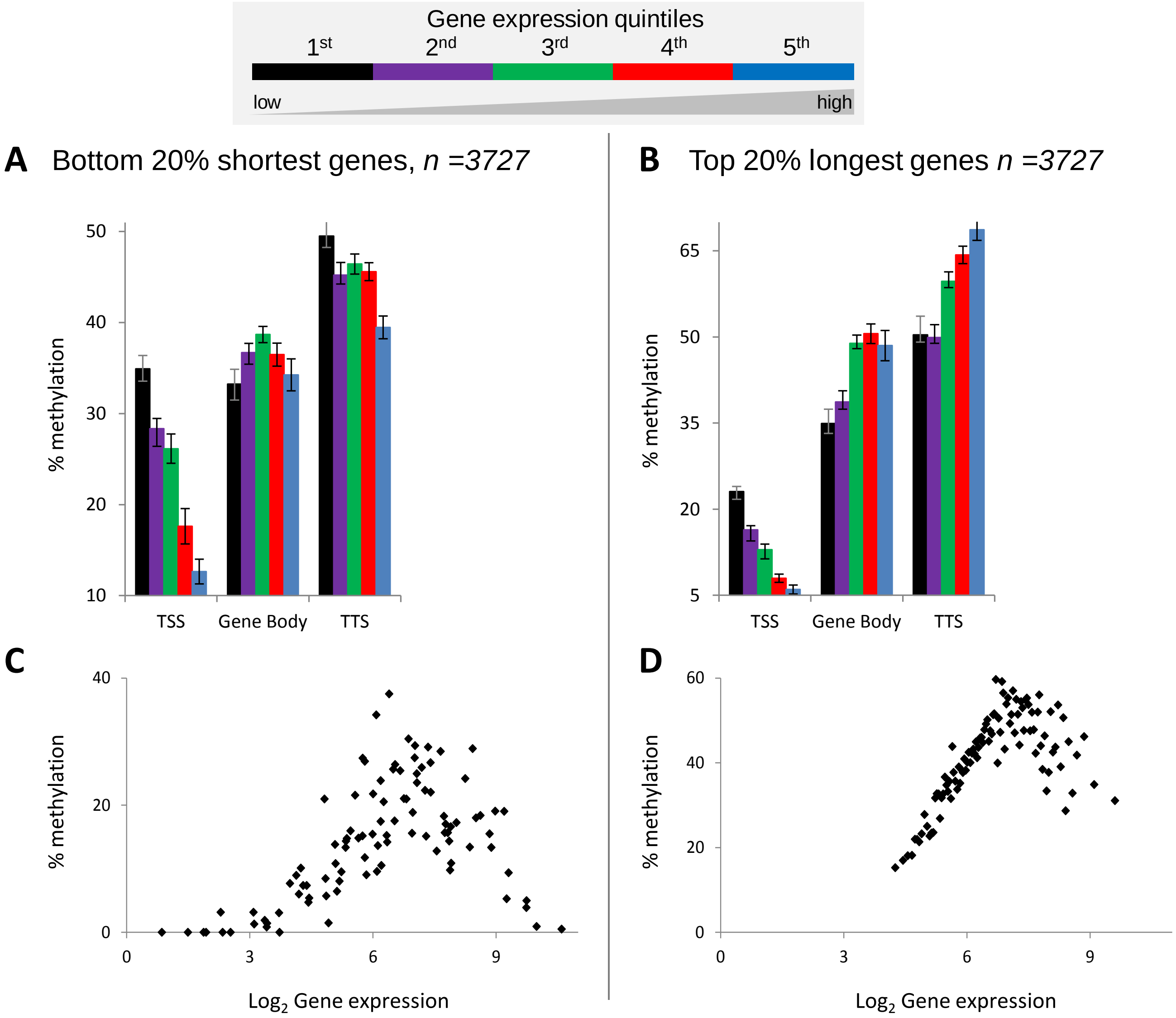

Supplementary Figure S3 – The bell shaped relationship between gene-body DNA methylation and gene expression is independent of gene length. Methylation levels for 5 gene expression bins at the TSS, gene-body and TTS for the 20% shortest (A) and 20% longest (B) genes. Relationship between gene-body DNA methylation and gene expression for 100 gene expression bins in the 20% shortest (C) and 20% longest (D) genes. All analysis performed in the GM12878 cell-line.

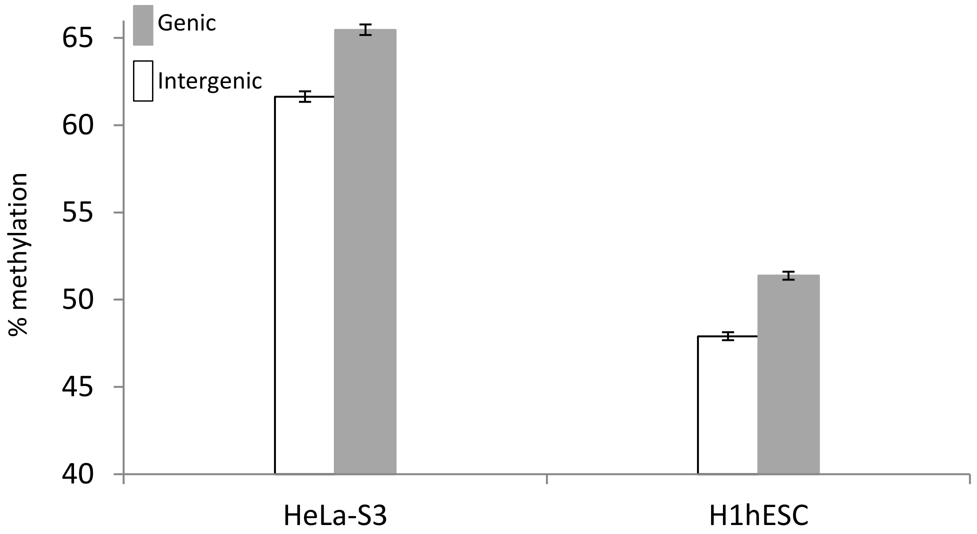

Supplementary Figure S4 – Comparison between genic and intergenic DNA methylation levels in HeLa-S3 and H1-hESC cell-lines. Error bars are standard errors.

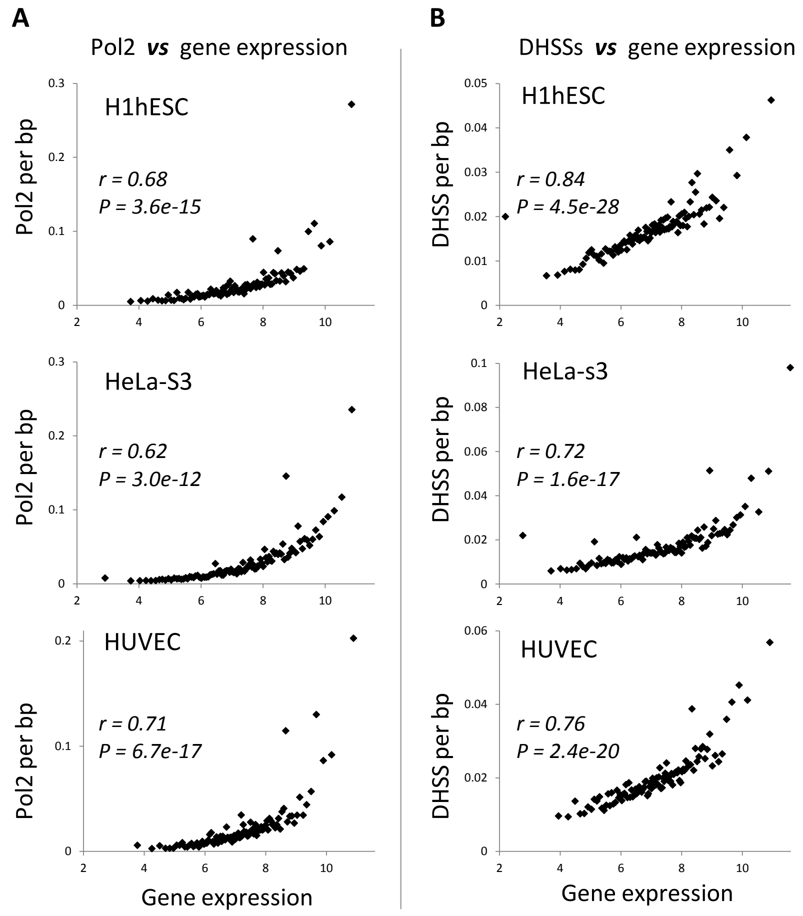

Supplementary Figure S5 – Relationship between gene expression and (A) Polymerase II density and (B) Density of DNaseI hypersensitive sites. Each data point represents the average Pol2 or average DHSS and the corresponding average gene expression of a bin of genes. Bins of genes are ordered by their average gene expression level. Pearson correlation coefficient values (*r*) along with their significance values (*P*) are shown for all pairwise regressions.
